# Database Mining Reveals Distinct Clusters of Putative Diphthamide-Specific Mono-ADP-Ribosylating Toxins in *Vibrio* and *Aeromonas*

**DOI:** 10.3390/toxins18100416

**Published:** 2026-09-28

**Authors:** Marta Mira Jørs, Geoffrey Masuyer, René Jørgensen

**Affiliations:** 1 Department of Science & Environment, Roskilde University, Universitetsvej 1, 4000 Roskilde, Denmark; 2 Department of Biochemistry and Biophysics, Stockholm University, Svante Arrheniusväg 16 C, 10691 Stockholm, Sweden; geoffrey.masuyer@dbb.su.se

**Keywords:** *Aeromonas* exotoxins, pseudomonas exotoxin A, PE-like toxins, PLT, *Vibrio cholerae* cholix toxin, mono-ADP-ribosylating toxins, evolution

## Abstract

Diphthamide-specific mono-ADP-ribosylating toxins form a structurally conserved but phylogenetically diverse family of AB-type bacterial toxins, with *Pseudomonas* exotoxin A (PE) serving as a prototypical member. Here, we expand the known diversity of PE-like toxins (PLTs) through comprehensive genome mining and phylogenetic, structural and sequence analyses of PE, Chx, and their *Aeromonas* and *Vibrio cholerae* homologues. *Aeromonas* toxins separate into two distinct groups: a PE-like cluster (AEI) and a cholix-like cluster (AEII), while a fourth cholix subgroup (ChxIV) is identified within *V. cholerae*, expanding the previously described ChxI–III classification. Although all newly identified toxins retain the core catalytic architecture and hallmark residues essential for ADP-ribosylation of eEF2, they differ in key functional motifs, including receptor-binding residues, furin cleavage loops, and C-terminal ER-retrieval signals, suggesting variation in host-cell uptake routes and intracellular trafficking. Signal peptides were present in all toxins but varied modestly between clusters, consistent with normal species-level Sec-pathway variation rather than divergent secretion mechanisms. Together, our findings provide a significantly expanded overview of PLT diversity and indicate that *Aeromonas*-derived toxins comprise at least two evolutionarily and mechanistically distinct groups. These insights offer a framework for future functional studies and highlight PLTs as emerging virulence factors in environmentally and clinically relevant bacteria.

## 1. Introduction

It is well established that exotoxin A (PE) from *Pseudomonas aeruginosa*, diphtheria toxin (DT) from *Corynebacterium diphtheriae*, and cholix toxin (Chx) from *Vibrio cholerae* are members of the diphthamide-specific AB class of mono-ADP-ribosyl transferases (mART) called the DT group toxins [1]. All three DT toxins have been shown to specifically target the post-translationally modified diphthamide residue of eukaryotic ribosomal elongation factor 2 (eEF2), which is essential for protein synthesis. Although these toxins share the same enzymatic target (eEF2), DT’s domain arrangement and cellular trafficking differ fundamentally from the Pseudomonas-like toxins. Diphtheria toxin carries its catalytic domain at the N-terminus and binding domain at the C-terminus, whereas PE, Chx, and related exotoxins have the reverse domain organization. These differences underlie distinct uptake and intracellular routing mechanisms.

Recent studies have further expanded the diversity of diphthamide-targeting mono-ADP-ribosylating toxins with the identification of additional homologues in environmental and opportunistic bacterial species [2,3]. Structural and biochemical characterization of these proteins confirmed that these toxins retain the conserved catalytic architecture and mechanism characteristic of PE and Chx; hence, they were presented as PE-like toxins (PLTs). PLTs consist of three structural domains: an N-terminal receptor-binding domain (domain I), the parts of which together form a 13-stranded anti-parallel β-jellyroll; a translocation domain (domain II) consisting of a bundle of six α-helices; and a C-terminal catalytic domain (domain III) with an α/β-fold topology [2,3,4,5]. A minor β-sheet segment sometimes labeled ‘domain Ib’ in the earlier literature is here considered part of domain II, as domain I alone suffices for receptor-binding/transcytosis.

In addition to the three structural domains, the PLTs contain an N-terminal signal peptide for secretion into the extracellular medium via a conserved two-step mechanism in Gram-negative bacteria. For example, PE has been shown to contain a 25 aa hydrophobic signal peptide. After expression, the precursor PE is transported from the cytoplasm to the periplasmic space using the Sec machinery [6,7] During translocation, the N-terminal signal peptide is cleaved off and PE is released into the periplasmic space. In the hydrophilic environment of the periplasm, PE is folded into its mature conformation in a manner that can be recognized by the type II secretion system (T2SS), specifically called Xcp in *P. aeruginosa*, for secretion into the extracellular space [8,9]. During the secretion by Xcp, two N-terminal acidic residues (+2 and +3) of the mature PE were found to be important both for efficient processing of the precursor protein and for extracellular localization of the toxin [10]. This is supported by another study showing that the first 30 N-terminal amino acids of the mature toxin are important for secretion along with parts of the C-terminal as well [6,8,11].

To reach its cytosolic destination of the host cells, the toxins first bind to a surface receptor of target cells, which for PE and Cholix toxins has been shown to be low-density lipoprotein receptor-related protein (LRP1) [4,12]. Receptor-binding is followed by receptor-mediated endocytosis upon which a lowered endosome pH triggers cleavage of a furin protease recognition site that is strictly conserved in domain II of the toxins.

After furin cleavage and reduction of an encompassing disulfide bond, PE has been shown to be retrogradely transported through the late endosome to the trans Golgi network. In the Golgi, the furin-generated carboxy-terminal fragment of PE, which includes the enzymatic domain, interacts with KDEL receptors via binding of its REDL C-terminal signal sequence, resulting in the toxin’s retrograde transport to the endoplasmic reticulum (ER). In the ER, the furin-cleaved toxin fragment undergoes partial unfolding and is exported to the cytosol by retro-translocation [13]. 

Once inside the host-cell cytoplasm, the DT toxins covalently transfer the ADP-ribose moiety from nicotinamide adenine dinucleotide (NAD^+^) onto the nucleophilic diphthamide imidazole on eukaryotic elongation factor 2 (eEF2) to efficiently inhibit protein synthesis and thus cause death of the host eukaryotic cells [4,14,15,16,17,18,19].

Curiously, while both DT and PE are secreted by known bacterial human pathogens, Chx was first identified in the non-pathogenic *V. cholera* TP strain from aquatic samples collected off the Californian coast, USA [20]. Chx are predominantly secreted by non-O1/non-O139 *V. cholerae* strains (or non-agglutinating *V. cholerae* (NAG)) that do not produce the two major virulence factors, cholera toxin (CT) and toxin-regulated pilus (TCP), mainly responsible for the acute and deadly diarrheal disease cholera, which affects millions of humans with inadequate access to clean water and sanitation facilities [21].

NAG *V. cholerae* strains exist in excess in the aquatic environment, and although a role for Chx in causing diseases in humans has not been clearly proven, several reports document that clinical isolates of non-O1/non-O139 strains positive for Chx cause diarrhea and extraintestinal infections in humans [22,23,24,25,26]. Infections with NAG are causing a wide clinical spectrum of gastroenteritis and extra-intestinal invasive disease, including cholera-like disease, bloody diarrhea, ear and wound infection (more frequently transmitted by seawater exposure) [27], meningitis, and bacteremia (predominantly in immunosuppressed patients) [28].

In support of this, Purdy et al. [29] assessed the prevalence of the *chxA* gene coding for Chx among a collection of 155 diverse *V. cholerae* clinical and environmental strains that originated in various countries, including Bangladesh and Mexico. They found that nearly one-half of 83 NAG strains and 16% of the 72 O1/O139 strains harbored a *chxA* gene. They suggested that Chx could play a role in the fitness of *V. cholerae* and that it may promote parasitic or mutualistic associations with aquatic dwelling eukaryotes. Furthermore, they suggested that Chx may enhance virulence during inflammatory gastrointestinal diseases in humans by combining with other virulence genes carried by NAG strains in which *chxA* is most prevalent.

TCP and CT are characterized as the main common virulence factors of *V. cholerae* O1 and O139, yet most of the NAG strains are devoid of both factors. Hence, NAG strains seem to cause disease in their host and survive in an ecological niche by different strategies than O1 and O139 serogroups [30,31]. Genomic mining studies suggest that NAGs may capture virulence factors through horizontal gene transfer and hence become toxic [20,28,31]. The potential of Chx as an important virulence factor in non-O1/non-O139 *V. cholerae* strains was further demonstrated by Awasthi et al. in 2013 [32]. In this study, 765 clinical *V. cholerae* strains were screened, of which 196 were non-O1/non-O139, and 53 of those (27%) were *chxA*-positive. They found that the identified *chxA* genes could be grouped into three clusters designated ChxI, ChxII, and ChxIII, of which ChxI and ChxII were shown to have cytotoxic effects on different eukaryotic cells. These findings thereby raise the possibility that Chx contributes to human disease.

In 2014, a putative PE-like exotoxin (AE) was identified in the Gram-negative *Aeromonas hydrophila* isolated from a clinical case of necrotizing fasciitis [33], and this AE was later suggested to be one of the main virulence factors promoting pathogenesis [34]. *Aeromonas* spp. are often found in aquatic environments and are known to cause furunculosis and hemorrhagic septicemia in fish such as carp, tilapia, perch, catfish, and salmon; red sore disease in bass and carp; and ulcerative infections in catfish, cod, carp, and goby [35]. This frequently leads to high mortality and severe economic losses in aquacultures [36,37].

However, an increased resistance to water treatment and antibiotics has also made certain *Aeromonas* species emerging human pathogens [38]. They are responsible for a range of human diseases that include intestinal and extraintestinal pathologies such as acute gastroenteritis and skin and soft tissue infections, but more systemic conditions such as septicemia and meningitis have also been reported [36,39]. Recently, AE was shown to be conserved across several pathogenic *Aeromonas* species, and a 2.3 Å crystal structure was solved for a catalytically inactive mutant from *Aeromonas hydrophila* [3]. The AE structure showed a high structural homology to the PE and cholix toxin structures as well as conservation of key residues such as disulfide bridges, the furin cleavage site and a C-terminal signal peptide responsible for retrograde transport to the endoplasmic reticulum of intoxicated host cells, and critical catalytic residues in domain III [40]. Hence, although the cytotoxic activity of AE is yet to be determined, it is likely to promote bacterial infection by inhibiting protein synthesis and causing cell death, thereby constituting a fourth member of this toxin family.

In this study, we expand the group of PLTs by thoroughly searching the NCBI database for homologues of previously described PE and Chx not originating from strains belonging to *P. aeruginosa* or *V. cholerae*. The searches revealed an abundance of homologues from different *Aeromonas species*, some of which were described in Masuyer et al.’s 2020 study [3], as well as new homologues from *Vibrio cholerae*. Interestingly, the homologues from Aeromonas species can be divided into two clusters, one with high homology to PE (AEI) and one with high homology to cholix toxin (AEII). The database search also revealed a new cluster of *chxA* gene sequences encoding a protein with very high homology to the AEII *Aeromonas* cluster. This new Chx cluster (ChxIV) is in addition to the three Chx clusters (ChxI–III) previously described [32], of which only ChxI and -II were shown to be cytotoxic on a selection of human, monkey and mouse cell lines.

A careful annotation of the newly identified sequences reveals that while the sequences in all clusters show conservation of some of the most crucial residues for toxin activity, there are also some differences between the clusters which could suggest the use of different approaches to intoxicate host cells and contribute to virulence. In addition, three-dimensional modeling by AlphaFold of the sequences in the newly found clusters shows high structural conservation in comparison to the previously solved structures of PE, ChxI and AEI, supporting that AEI, AEII and ChxIV all have the potential to be active exotoxins. However, the activity, host-cell target and potential virulence patterns of these newly identified variants are yet to be confirmed.

## 2. Results

### 2.1. Genome Mining

The NCBI non-redundant (nr) protein sequence database was screened to identify homologues of the PE sequence from *Pseudomonas aeruginosa*, using PSI-BLAST. Two iterations of PSI-BLAST search were performed with the query sequence WP_058170169.1. The results were filtered to include only those results that had both a high query coverage (≥90%) and a high percent identity (≥40% identity to PE). No new sequences were identified above the 0.0001 threshold in the second iteration. The search identified an overwhelming number of potential toxin sequences from *Aeromonas* spp., of which seven were assigned standard binomial species names (Table 1). In total, after the second iteration and application of the filtering criteria, the search yielded 398 hits.

The hits were sorted according to their percentage identity to PE. For the *Aeromonas* spp. with standard binomial names, the sequence showing the highest percentage identity to PE was selected. The corresponding accession numbers and other relevant information from the search are presented in Table 1. The search was then repeated with the PE catalytic sequence fragment as the query to determine the percentage identity of the catalytic domain within the selected sequences. The selected sequences all had a sequence identity between 56 and 65% and an almost full coverage to PE. Furthermore, the *Aeromonas* sequences had E-values below 1 × 10^−180^, and hence there was a limited risk that the alignment occurred by chance. When comparing only the catalytic domains (domain III) of the *Aeromonas* sequences to PE, the sequence identity was significantly higher (>70%), supporting that these *Aeromonas* domain IIIs likely have retained their catalytic activity and diphthamide-specific target on eEF2.

The NCBI non-redundant (nr) protein sequence database was also screened to identify sequences homologous to ChxI from *V. cholerae*. A PSI-BLAST search was performed using the *V. cholerae* ChxI query sequence Q5EK40.1. After the third iteration, no new sequences were found above the 0.0001 threshold. The results were then filtered to include only sequences with high query coverage (≥90%) and high percent identity (≥40%), resulting in six hits. These hits did not appear in the previous PSI-BLAST results using PE as the query and included AE sequences from *A. salmonicida* and *A. popoffii*, as well as one sequence (WP_416246183.1) annotated as belonging to multiple species, including *A. veronii*. Two accession numbers representing two species with standard binomial names were selected based on their percentage identity to ChxI and are shown in Table 2. As for the PE search, Table 2 also includes the percentage identity of the catalytic domain, obtained through an additional search using the ChxI catalytic sequence fragment as the query. The two selected sequences both show almost full query coverage against ChxI and E-values below 1 × 10^−180^. Overall, the ChxI-like AE sequences (AEII) showed lower percentage identities than the PE-like AE sequences (AEI) presented in Table 1. Both the full-length sequence identity and the catalytic domain sequence identity were approximately 50%. This relatively high sequence identity, particularly within the catalytic domain, provides an indication that these *Aeromonas* proteins may have retained the catalytic activity and the ability to target the diphthamide residue of eEF2. It is worth noting that although the AE hits retrieved from the two PSI-BLAST searches were distinct, representatives of both AE types were found to co-occur in some genomes, with the corresponding AEI and AEII sequences located on different contigs (Supplementary Table S1). Sequences retrieved from the two PSI-BLAST searches above, combined with the query sequences, were aligned and used to construct a phylogenetic tree comprising 404 AE sequences in total (Supplementary Figure S1).

Finally, a PSI-BLAST search was performed using the *A. popoffii* hit identified in the previous search (accession number WP_212514733.1) as the query. The NCBI nr database was searched for Chx variants from *V. cholerae* with higher sequence similarity to AE than ChxI (Q5EK40.1). After the third iteration, no new sequences were found above the 0.0001 threshold. The results were then filtered to include only sequences with high query coverage (≥90%) and high percent identity (≥40%), resulting in three hits with almost full coverage and E-values below 1 × 10^−180^. Importantly, all three newly identified potential Chx sequences were clearly distinct from the ChxI-III clusters previously identified by Awasthi et al. in their 2013 study [32]. Compared to the AEII from *A. popoffii*, the sequences in this new Chx cluster, hereafter referred to as ChxIV, showed high sequence identity, with approximately 83% identity across the full-length sequence and 85% identity within the catalytic domain (Table 3). This high degree of sequence conservation makes it likely that ChxIV retains features associated with catalytic activity and recognition of the dipthamide residue of eEF2.

To further investigate the relationships between the sequences identified through the BLAST searches described above, all selected AE sequences shown in Table 1 and Table 2 were aligned together with the previously described PE sequence, selected ChxIV sequences from Table 3, and representatives of ChxI-III described by Awasthi et al. in 2013 [32]. The sequence alignment generated using COBALT was used to construct a distance-based phylogenetic tree (Figure 1). The tree visualizes the clustering of the studied sequences and illustrates that the Chx-like AE sequences, shown in Table 2, constitute a distinct cluster closely related to the ChxIV cluster. This led us to name the *Aeromonas* sequences showing homology to PE and Chx for AEI and AEII, respectively. The phylogenetic analysis further indicates that the AEI sequences group closely with PE, suggesting a Pseudomonas-like lineage, whereas AEII sequences cluster with the Chx variants, particularly the newly identified ChxIV, forming a distinct branch. This phylogenetic split implies at least two separate acquisition events or ancestral origins for *Aeromonas* exotoxins: one more related to *Pseudomonas* PE and another to *Vibrio* Chx. Such a scenario is consistent with horizontal gene transfer (HGT) playing a role in disseminating these toxin genes across genera, as has previously been reported for other PLTs [2].

### 2.2. Sequence Alignment

The protein sequences were analyzed in a multiple structural alignment (MSA) using either crystal structures or AlphaFold models of representative sequences from each of the clusters shown in Figure 1. The MSA shows several fully conserved patches throughout all three domains in all representative sequences (Figure 2). The eight conserved Cys residues are present in all clusters, including one pair that potentially forms a disulfide bridge spanning the furin cleavage site. In addition, the previously described residues in domain III (His, Tyr, Tyr, Glu, and Glu), which are known to be crucial for catalytic ADP-ribosyl transferase activity, are fully conserved.

### 2.3. Signal Peptide Sequences

N-terminal signal peptides direct bacterial proteins for secretion into the extracellular medium. In the Sec-dependent pathway, the signal peptide targets the exported protein to the SecYEG-complex, and it is then transported through the Sec channel in an unfolded state. Out of the total of 432 analyzed sequences (1 PE, 398 AEI, 6 AEII, 12 ChxI, 9 ChxII, 3 ChxIII, and 3 ChxIV sequences), 425 were classified by SignalP 6.0 [41] as containing a Sec/SPI signal peptide, with predicted Sec/SPI probabilities ranging from 0.501 to 0.999. Among the 425 sequences classified as containing a signal peptide, Sec/SPI was the only signal-peptide type predicted. Thus, although the Sec-dependent secretion pathway was consistently predicted, the confidence of the Sec/SPI classification varied among sequences, particularly within the AEI group, which included several borderline predictions. With the exception of the shorter AEI sequences, all groups had predicted probabilities of 0.999. Supplementary Table S2 shows all unique signal peptide sequences identified among the 425 sequences classified as containing a signal peptide, while Figure 3 summarizes their sequence composition as sequence logos.

Due to the large number of AEI sequences and the presence of both short (11–13-amino acid-long) and long (26-amino acid-long) N-terminal sequences, these two groups were analyzed separately. The longer AEI group contained the conventional 26-residue signal sequence, and it was represented by 38 accession numbers, whereas the shorter group, represented by 353 accession numbers, contained a 13-residue region. By further exploring the GenBank DNA sequences, we discovered that these short sequences, at least in some cases, likely represent truncated GenBank records. Supplementary Figure S2 shows selected AEI signal peptide regions together with extended versions reconstructed using upstream genomic sequences. These extended sequences were similar or identical to the long AEI signal peptide shown in Figure 3. Addition of the upstream sequence resulted in substantially stronger Sec/SPI predictions, supporting the possibility that some of the short AEI records are incomplete.

Although a Sec-type signal peptide was predicted across all protein groups, the precise signal peptide sequences varied considerably between clusters (Figure 3). The PE signal peptide showed no obvious conservation with the AEI signal peptides, which themselves fall into two distinct groups that were mentioned earlier. The AEII and ChxIV signal peptides were highly conserved with each other, yet clearly distinct from the ChxI -III signal peptides, which form their own conserved group. Such variation in signal peptide composition is common among bacterial secretory proteins and most often reflects species- or lineage-specific optimization rather than changes in secretion mechanism [42]. Accordingly, we interpret these differences as being consistent with the phylogenetic separation of the clusters rather than evidence for altered export pathways.

The structural alignment also revealed that AEI toxins contain the lysine residue in domain I (K69 in AEI, corresponding to K57 in PE) that was previously shown to be implicated in binding to the LRP1 receptor of the host cells (Figure 2, Figure 4 and Figure 5), as replacing this residue disrupts receptor binding [12,43,44]. Hence, it is likely that AEI shares the same LRP1-dependent intoxication route as PE. At the corresponding position in the β4 position of the β-jellyroll fold, the AEII and ChxIV have a threonine residue, whereas ChxI, -II and -III have an isoleucine, none of which share the positive charge of lysine. ChxI was previously shown to be toxic to an LRP-deficient strain of mouse embryonic fibroblast cells; however, not to the same extent as for normal (LRP^+/+^) mouse embryonic fibroblast cells [4]. This suggests that ChxI may exploit alternative avenues, besides the LRP receptor, for entering the host-cell cytoplasm. However, additional experiments are required to confirm the role of LRP1 or identify other potential cell surface receptors for ChxI to -IV as well as for AEII.

### 2.4. Furin Cleavage Site

It is well established that the group of diphthamide-specific ADP-ribosylating toxins are activated within host cells by cleavage of an arginine-rich loop followed by reduction of a critical disulfide bridge spanning the cleavage site [13,45,46]. This Arg-rich motif (R^274^HRQPRG^280^ in PE) is recognized by the protease, furin, which is shown to cleave PE between the residues R^279^ and G^280^. The structural alignment shows that AEI has a furin cleavage motif (RSRQPRG) that is highly conserved to the motif in PE, while AEII instead has a motif that is identical to ChxI and -II (RSRKPRD), which are known to be cytotoxic (Figure 2 and Figure 5) [4,32]. On the other hand, ChxIII has a slightly modified furin cleavage motif (RGRKPRD), and in a recombinant version of this toxin it has been shown that the replacement of the serine with glycine hampers the proteolytic activity of furin [32]. This may explain the reason for ChxIII failing to induce cytotoxicity in a series of tested cell lines. The newly identified ChxIV has a motif (RSRDELR) that shows limited conservation compared to the known furin cleavage sites, and it remains to be determined if this motif is recognized and activated by the furin protease.

### 2.5. Retrograde Transport to the ER

The C-terminal signal peptide (R^609^EDL^612^K) of PE has been shown to bind to the KDEL receptor, upon which the toxin is transported to the endoplasmic reticulum in a retrograde manner. However, PE first needs to have its carboxy terminal lysine removed by an extracellular carboxypeptidase that reveals the REDL signal sequence [47].

AEI possesses a KDEL-like C-terminal sequence (RDEL) similar to that in PE but closer to the preferred consensus motif for KDEL receptor binding and without a C-terminal lysine. In fact, a mutated version of PE with an RDEL motif in the C-terminus showed an increase in cytotoxicity compared to REDL, suggesting that AEI might be more efficient at entering the cytoplasm via the Golgi apparatus [48].

Both ChxI and -II have a KDELK which, as for AEI, may also facilitate a higher affinity for the KDEL receptor, assuming that the C-terminal lysine is removed as for the REDLK motif in PE. It has also been shown that replacing the REDL in PE with a KDEL increases the cytotoxicity of PE, and therefore ChxI and ChxII may also be more efficient at entering the cytoplasm [49,50]. ChxIII has an HDEL sequence instead of KDEL. A receptor present in human cells has been shown to bind toxins with both KDEL and HDEL sequences in vitro [51,52]. In addition, HDEL receptors are found in both *Saccharomyces cerevisiae* and plants, suggesting that ChxIII could have functional capabilities to target a different host system [51,53,54]. Finally, neither ChxIV nor AEII contain a carboxy terminal KDEL receptor-binding motif. Whether this disrupts their transport into the cytoplasm, thereby making the toxins non-functional, or whether these two variants utilize another trafficking route still needs to be determined.

### 2.6. Structural Comparison

The crystal structures and AlphaFold models used for the MSA reveal an overall conserved core fold across all clusters (Figure 4), with pairwise Cα RMSD values of 1.7–2.8 Å between experimentally solved structures (PE vs. AEI; PE vs. ChxI) and 2.0–5.5 Å among the ChxII/ChxIII AlphaFold models and their closest experimental relative (ChxI). Across every pairwise comparison, including between the two experimentally solved structures, PE and AEI, the region encompassing the furin cleavage loop and adjacent domain consistently shows the largest local Cα deviations (up to ~19 Å), indicating that this is a genuinely flexible region of the fold conserved across the toxin family rather than an AlphaFold-specific artifact. In the ChxII model, this is reflected in reduced local confidence for the furin cleavage loop itself (residues 282–287; average pLDDT = 32.1 vs. 73.0 for the full model), which could potentially allow easier access for proteolytic processing. The conserved cysteine pair (Cys273–Cys295), which may form a disulfide bridge, falls within this same divergent region and is displaced by ~14–17 Å relative to the corresponding residues in the ChxI structure (Figure 4D). Given the inherent uncertainty in AlphaFold-modeled loop conformations and the recurrent flexibility of this region across both modeled and experimentally solved structures, we limit our interpretation to these two observations rather than drawing broader mechanistic conclusions.

All toxins in the PLT family share a β-jellyroll receptor-binding domain (domain I) with a key exposed loop. In PE, a lysine residue on β-strand 4 (Lys57) is critical for binding LRP1 on host cells. We observe that this Lys (or an analogously positioned basic residue) is present in most AEI sequences and some AEII/cholix variants but is substituted in others (Figure 2). The absence of a positively charged residue at this site in certain toxins (e.g., ChxII/III) might indicate altered receptor usage. For example, ChxI is known to engage alternative receptors like TMEM132A for cell entry in epithelial tissues [55]. This sequence divergence in the LRP1-binding motif suggests that different PLT family members may exploit distinct host receptors or cell tropisms, which will require future experimental validation.

Most examined toxins retain the canonical, or near-canonical, furin cleavage sequence (e.g., RXXR in domain II), consistent with the need for host-mediated proteolytic activation. However, we note that sequences immediately adjacent to the cleavage site differ between clusters. Previous observations reported that minor variations in the loop adjacent to the furin motif can alter cleavage efficiency [2]. For example, certain PLTs (Hmx and Jlx) possess a unique ‘K-D-A’ triad near the furin site, correlating with more efficient cleavage at neutral pH, whereas a negatively charged residue in this position (as seen in others) was associated with slower activation. Although we have not tested cleavage kinetics here, the sequence differences in this ‘activation loop’ among AEI, AEII, and Chx toxins raise the possibility of differential furin processing rates in vivo, potentially influencing how quickly each toxin can exert its effect.

Additionally, comparison of the catalytic domains (domain III) across all clusters showed a high degree of structural conservation. The core α/β-fold characteristic of diphthamide-specific ADP-ribosyltransferases was preserved in AEI, AEII and all Chx variants, and the key catalytic residues previously shown to be essential for NAD^+^ binding and ribosyl transfer were fully conserved. Subtle sequence differences were observed in peripheral loops surrounding the active site, but none seem to physically disrupt the conserved catalytic pocket. This overall conservation supports that all identified putative toxins, regardless of lineage, are likely to retain canonical ADP-ribosyltransferase activity toward eEF2, consistent with the strong conservation observed in the sequence alignment (Figure 2).

Figure 5 shows an overview of the key functional sites and motifs in the different toxin variants. The figure underscores that while all toxins share the essential hallmarks for activity, the LRP-binding site (when present), the furin cleavage motif, and the diphthamide-targeting catalytic residues, there are notable differences that could modulate each toxin’s mechanism of action. For instance, the presence of a C-terminal KDEL or variant thereof varies across clusters: most PE/AEI-type toxins possess a KDEL-like ER retrieval signal (e.g., REDL in AEI), whereas AEII and ChxIV lack this motif. As discussed below, this could mean that some of these newly identified toxins rely on alternative routes (or host factors) to reach the cytosol, an aspect that warrants further investigation.

### 2.7. Evolutionary History

To evaluate whether these toxin genes might be horizontally acquired, we compared their GC content to that of their host genomes. Notably, the *Aeromonas* (AEI) toxin genes do not markedly deviate from the genomic GC% (e.g., AEI gene ~61% GC vs. ~60–62% for *A. hydrophila* core genome), suggesting they have had sufficient time to ameliorate or a common lineage within Aeromonas. By contrast, the AEII gene from *A. salmonicida* (GenBank accession WP_290077894.1, contig NZ_JAOXBX010000039.1) exhibits a coding DNA sequence GC content of only ~41%, some 18 percentage points below the ~58.5% genomic average reported for *A. salmonicida* strain A449 (56). Similarly, the ChxIV gene from *V. cholerae* strain 2015V-1093 (contig ABJSWY010000006.1) shows ~40% GC content, well below the *V. cholerae* genomic average of ~47% (57). Both toxin genes are notably depleted in GC content relative to their respective host genomes, consistent with horizontal gene transfer. This pattern is consistent with an independent analysis by Masuyer et al. (2025) [2], who found that several PLT genes—including those from *Serratia fonticola*, *Pseudomonas aeruginosa*, and *Vibrio cholerae*—show GC content and base-composition profiles that deviate from their host core genomes, supporting a broader pattern of horizontal acquisition across the PLT family. Although a rigorous comparative analysis of all cluster representatives is beyond the scope of the present study, the broad distribution of these toxins across disparate taxa (Figure 1) raises the possibility that horizontal gene transfer has contributed to their evolutionary history [2]. This possibility, however, remains to be established through genomic-context analyses.

## 3. Discussion

This phylogenetic and sequence analysis substantially expands our understanding of diphthamide-specific mono-ADP-ribosylating toxin (PLT) diversity [2,3]. Our findings delineate two evolutionarily distinct *Aeromonas* toxin clusters (AEI and AEII) and identify a novel ChxIV subgroup within *Vibrio cholerae*, demonstrating that PLT diversity far exceeds previous estimates. A key observation is that despite conservation of the catalytic mechanism across all identified toxins [4,5,14], significant variations exist in receptor-binding residues, furin cleavage loops, C-terminal trafficking signals, and signal peptide sequences. These differences suggest that individual toxins may have adapted to distinct ecological niches or host-cell types.

The signal peptide variation observed across AEI, AEII, and Chx clusters aligns with normal species-level variation in Sec-pathway secretion [6,42], supporting phylogenetic separation of the clusters rather than fundamentally altered secretion mechanisms. More notably, the absence of canonical C-terminal KDEL-like ER-retrieval signals in AEII and ChxIV is striking, given their essential role in PE and classical Chx trafficking for retrograde Golgi transport and subsequent cytosolic translocation [4,12]. Other PLT family members (Hmx, Jlx, and Sfx) similarly lack canonical KDEL motifs [2]. The evidence from diphtheria toxin (DT), which forms a membrane pore via its translocation domain to promote cytosolic release despite lacking KDEL [56], and from alternative pathogens such as Shiga toxin and ricin, which exploit distinct endosome-to-Golgi or membrane-poration pathways [57], suggests that AEII and ChxIV variants may circumvent the canonical PE-like trafficking route. Thus, these KDEL-lacking toxins warrant systematic experimental investigation to define their intracellular trafficking mechanisms.

The structural and sequence differences between AEI and AEII, particularly in domains governing receptor recognition and intracellular trafficking, imply that these toxins likely follow distinct cell-entry pathways and may display divergent intoxication profiles. This functional divergence is notable given that *Aeromonas* species occupy a remarkably wide range of aquatic and terrestrial ecological niches and cause diseases ranging from gastroenteritis to invasive infections in both aquaculture and clinical settings [36,38,39]. The presence of two mechanistically distinct toxin groups may reflect specialization for different host interactions or environmental pressures. Similarly, the diversification of *Vibrio cholerae* Chx subtypes in non-O1/non-O139 strains suggests adaptive specialization to alternative virulence strategies [30,31]. The identification of multiple Chx variants and their association with pathogenic non-O1/non-O139 strains highlights the role of HGT in shaping toxin diversity [29,32] across these important environmental pathogens.

From translational and clinical perspectives, our findings have implications for understanding emerging infections and for biotechnology development. *Aeromonas* species are increasingly recognized as causative agents of gastroenteritis, wound infections, and systemic disease, including meningitis and bacteremia in immunocompromised patients [27,28,39], while non-O1/non-O139 *Vibrio cholerae* similarly cause a spectrum of clinical manifestations [22,23,24,25,26]. The identification of putative exotoxin homologs in these organisms underscores the need for active surveillance of their potential contributions to virulence [34]. Furthermore, much like PE and diphtheria toxin, which have been successfully engineered into immunotoxins and vaccine delivery platforms [58,59], the newly characterized putative AE and Chx toxin variants (AEII and ChxIV) could be investigated as potential scaffolds for therapeutic applications. Whether these putative virulence factors could circumvent anti-toxin immunity or provide enhanced tissue-targeting properties warrants further investigation. Elucidating the functional properties and intoxication mechanisms of each PLT cluster will thus be crucial for both understanding their roles in bacterial pathogenesis and evaluating their translational potential.

## 4. Materials and Methods

### 4.1. Screening of New PE and ChxI Exotoxin Homologues in Aeromonas Species

The NCBI non-redundant (nr) protein sequence database was screened for PE-like and ChxI-like putative AE toxins by performing iterative BLASTp searches using Position-Specific Iterated BLAST (PSI-BLAST) in August 2026. The searches were constrained to *Aeromonas* protein sequences (taxid:642), while *Vibrio cholera* (taxid:666) and *Pseudomonas* (taxid:286) were excluded. The query sequences used were accession number WP_058170169.1 for PE and accession number Q5EK40.1 for ChxI. Unless otherwise stated, default PSI-BLAST parameters were used, including the BLOSUM62 substitution matrix. The Expect threshold value was set to 0.000001, while the PSI-BLAST Threshold was set to 0.0001, thereby reducing the likelihood of incorporating false-positive hits. PSI-BLAST searches were run until no new hits were identified, with the final iteration being the first iteration to yield no additional hits. The resulting hits were filtered by setting the query coverage range to 90–100% and the percent identity range to 40–100%. Furthermore, the two PSI-BLAST searches were repeated using only the catalytic domain fragments of the original query sequences. This was done to assess the sequence identity of the catalytic domains among the selected hit sequences.

### 4.2. Screening for New Chx Toxin Homologues in Vibrio Cholerae

The NCBI non-redundant protein sequences database was screened for AEII-like Chx sequences by performing an iterative PSI-BLAST search in August 2026. The AEII query sequence used was accession number MDM5064611.1, with the search constrained to *Vibrio cholerae* (taxid:666), while Aeromonas (taxid:642) and Pseudomonas (taxid:286) sequences were excluded. Otherwise, the search parameters and procedures were identical to those in the two PSI-BLAST searches described above.

### 4.3. Phylogenetic Tree of All Identified Aeromonas Exotoxin (AE) Sequences

The filtered hits obtained from the two PSI-BLAST searches using PE (WP_058170169.1) and ChxI (Q5EK40.1) were combined with the respective query sequences, resulting in a total of 404 protein sequences (402 hits and 2 query sequences). These protein sequences were aligned using the Constraint-based Multiple Alignment Tool (COBALT) [60] from NCBI with the default settings. A distance-based phylogenetic tree was then constructed in COBALT from the resulting alignment using the Fast Minimum Evolution method with the Grishin distance measure. The obtained tree was subsequently visualized and annotated using the Interactive Tree of Life (iTOL) in a circular layout, with branch lengths ignored. The sequences shown in Table 1 and Table 2 were also highlighted in the tree for reference. No branch support analysis was performed.

### 4.4. Phylogenetic Tree—PLT Clusters

To obtain an overview of the protein sequence diversity of PE, Chx and AE and the relationships between their distinct types, PE, together with selected representative sequences of Chx clusters (ChxI-IV) and AE clusters (AEI and AEII), was included in a COBALT alignment. A phylogenetic tree was then constructed in COBALT using the Fast Minimum Evolution method and Grishin distance measure. The so-constructed tree was subsequently visualized using iTOL. The rectangular layout was selected, with branch lengths retained.

### 4.5. Sequence Alignment

The PROMALS3D multiple sequence and structure alignment server was used to create alignment based on fasta sequences [61]. Structural coordinates for PE and ChxI were obtained from the Protein Data Bank with PDB entry codes, 1IKQ and 2Q5T, respectively. Structures of the remaining protein sequences (AEI and AEII from *A. salmonicida* and ChxII, ChxIII and ChxIV from *Vibrio cholerae*) were modeled using AlphaFold (version 2.3.2) [62] in the Google CoLab system [63]. The resulting alignment was then uploaded to ESPript3 for creation of the graphical output, including secondary structure annotations [64]. Finally, additional colored annotations highlighting critical residues in the sequence alignment were performed manually using Affinity Designer 2 (version 2.6.5) by Affinity Studio [65].

### 4.6. Signal Peptide Analysis

The machine-learning-based predictor model SignalP 6.0., developed by DTU Health Tech [41], was employed to predict signal peptides and their cleavage-site positions in a total of 432 protein sequences (1 PE, 398 AEI, 6 AEII, 12 ChxI, 9 ChxII, 3 ChxIII, and 3 ChxIV sequences). Accession numbers of all those sequences are shown in Figure 1 and Supplementary Figure S1. Sequences were screened for signal peptides using the slow mode of SignalP 6.0. Based on the SignalP 6.0 prediction summary, the predicted signal peptide fragments were further analyzed using R (R version 4.5.2) [66]. The R package ggseqlogo [67] was used to generate sequence logos to visualize the conservation and diversity of N-terminal sequences within and between the different putative and established toxin groups. PE, AEI, AEII, ChxI, ChxII, ChxIII, and ChxIV were analyzed separately. Thus, the sequence logos were used to illustrate patterns of sequence conservation and diversity within each group and differences between groups, rather than to infer evolutionary probabilities or phylogenetic relationships. For groups represented by a single unique signal peptide sequence, the logo represents the sequence composition rather than within-group conservation. Due to the large number of AEI sequences and the presence of both shorter and extended N-terminal sequences, AEI sequences were analyzed separately according to sequence length.

Because some toxin groups contained relatively few sequences, the custom mode of ggseqlogo was used to allow the omission of a default small-sample correction. For each alignment position, the information content was calculated as $R={log}_{2}(20)-H$, where H is the Shannon entropy of the amino acid frequency distribution. Individual amino acid letter heights were calculated as $p_{i}R$, where *p_i_* is the frequency of amino acid *i* at that position. Gaps were excluded from frequency calculations. The theoretical maximum information content for an amino acid position is ≈4.32 bits.

## Figures and Tables

**Figure 1:**
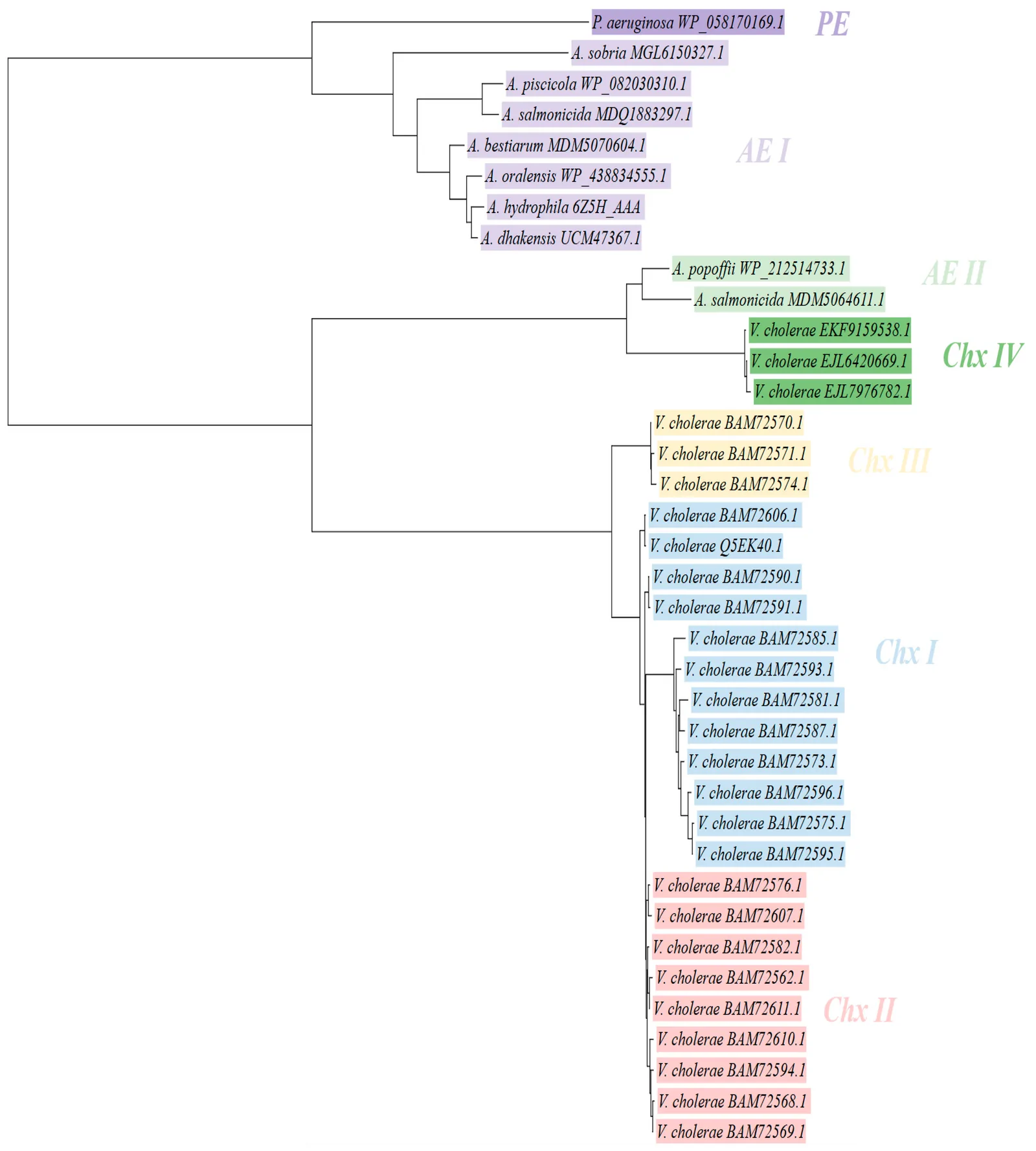
Phylogenetic tree showing four distinct Chx clusters (ChxI (light blue), ChxII, (light red) ChxIII (yellow), and ChxIV (dark green)), two AE clusters (AE I (light purple) and AE II (light green)) and PE. The AE I (*n* = 7), AE II (n = 2), and ChxIV (*n* = 3) sequences are the same as those identified in Table 1, Table 2 and Table 3, respectively. PE (WP_058170169.1) and ChxI (Q5EK40.1) were used as search queries in Table 1 and Table 2. The ChxI (*n* = 3), ChxII (*n* = 13) and ChxIII (*n* = 8) clusters were previously described in [32]. The tree was generated using the web-based programs COBALT and iTOL. Branch lengths represent the estimated evolutionary distances between sequences based on the Grishin distance measure. Colors are aligned with Table 1, Table 2 and Table 3.

**Figure 2:**
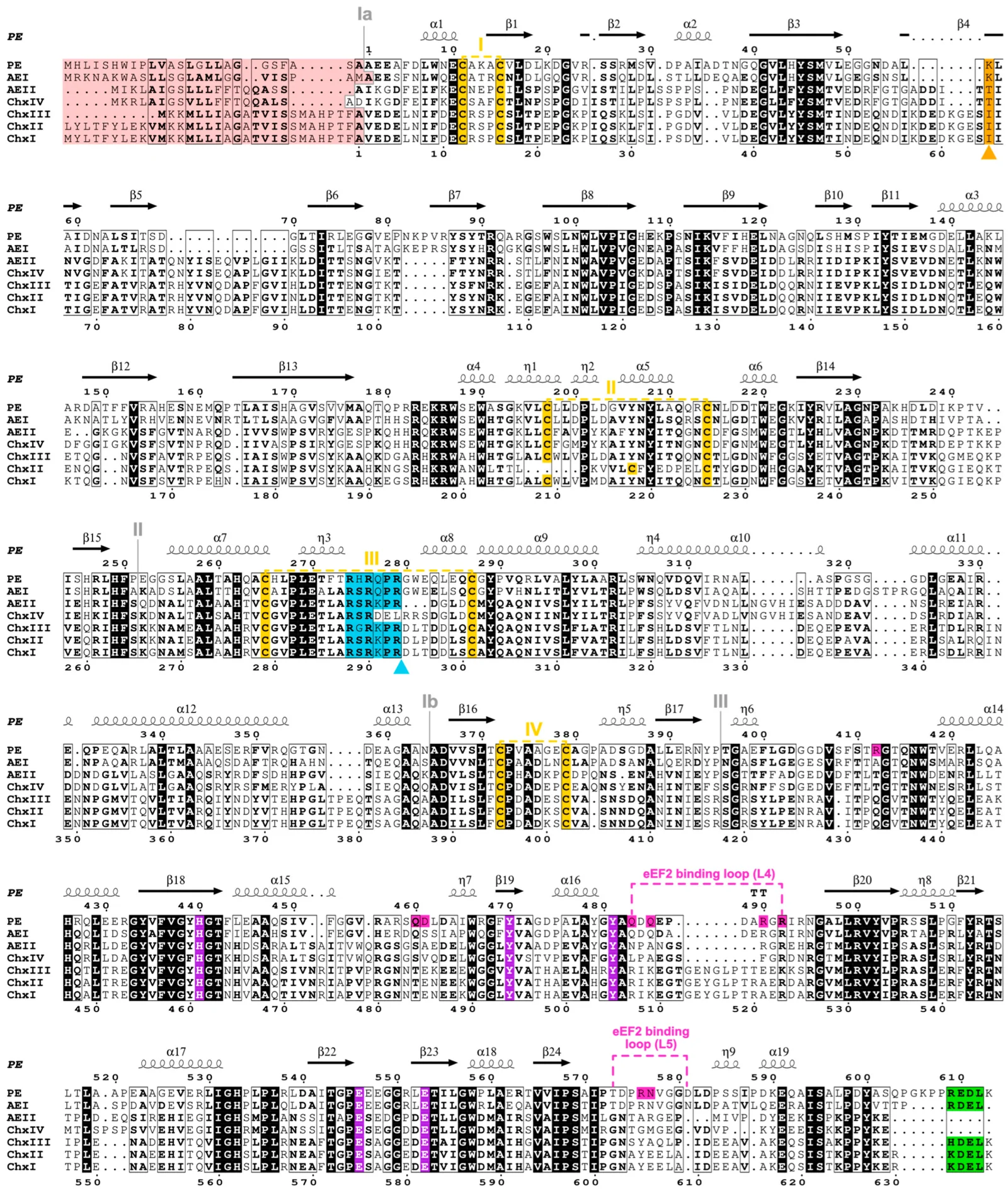
Multiple sequence alignment of *P. aeruginosa* PE (WP_058170169.1), representatives of AEI (MDM5061562.1) and AEII (MDM5064611.1) from *A. salmonicida*, and representatives of ChxI (Q5EK40.1), ChxII (BAM72585.1), ChxIII (BAM72570.1), and ChxIV (EKF9159538.1) from *V. cholerae*. Conserved positions are highlighted, and the secondary structure based on *P. aeruginosa* PE (PDB Entry 1IKQ) is plotted above the alignment. Predicted signal sequences are indicated in light red, and suggested disulfide bridges are shown in yellow and labelled with Roman numerals (I-IV). The lysine (K) important for LRP receptor binding is shown in orange. The furin motif is shown in cyan, with the cleavage site marked by a triangle. Conserved catalytic active-site residues are shown in purple, the RDEL retrograde transport C-termini are marked in green, and residues important for eEF2 binding, including the two eEF2 binding loops (L4 & L5), are shown in magenta. The beginnings of domains I, II, and III are marked in grey. Ia and 1b refer to the traditional domain organization. Residue numbering at the top and bottom of the alignment corresponds to the PE and ChxI sequences, respectively, and starts after the signal peptide.

**Figure 3:**
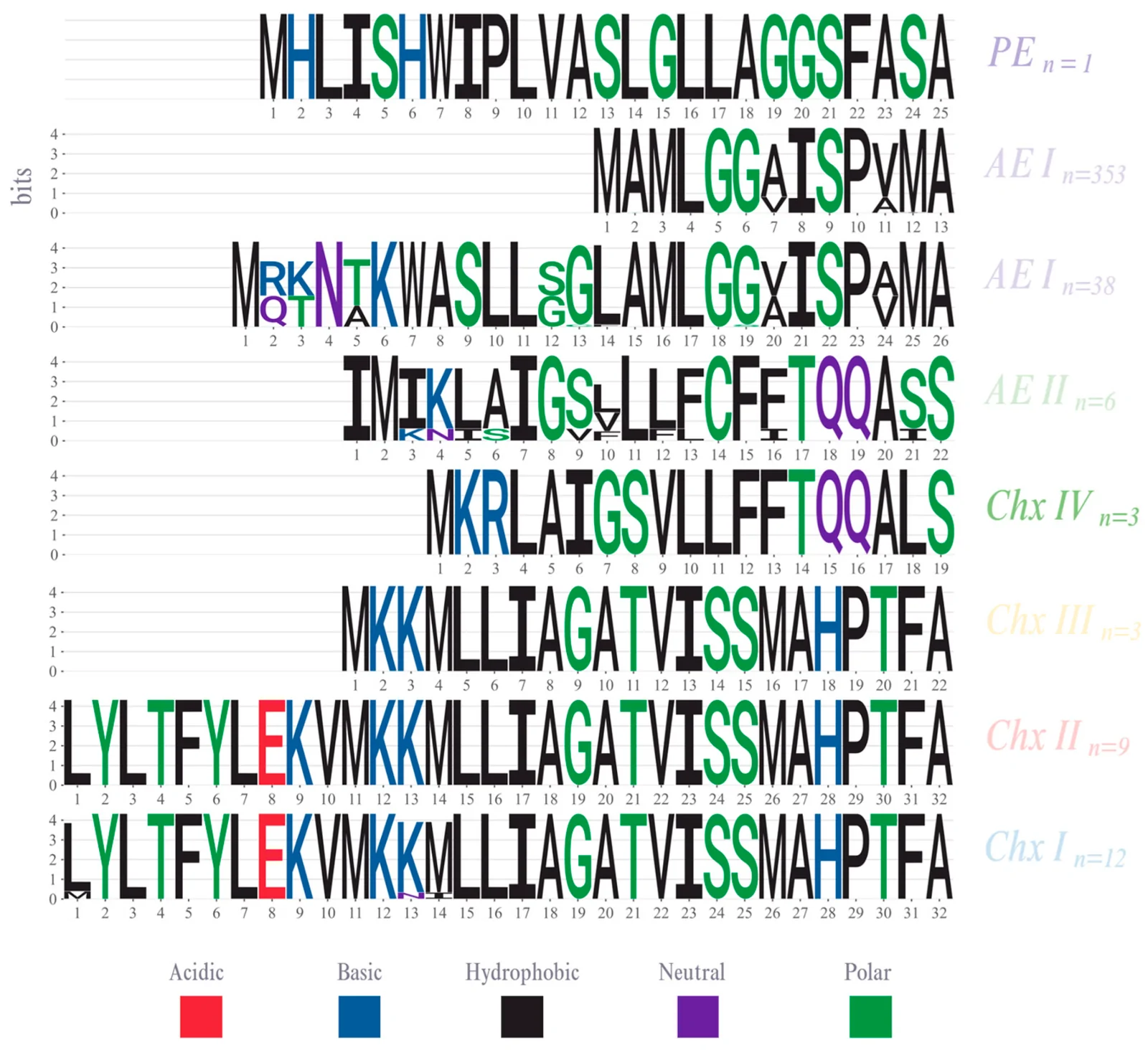
Sequence logos showing the amino acid composition of predicted Sec/SPI signal peptides across PE, AEI, AEII, and ChxI–IV protein clusters. The number of sequences used to generate each logo is indicated next to the corresponding cluster name as a subscript (*n* = x). Letter heights represent amino acid information content, with a maximum of 4.32 bits for a fully conserved position.

**Figure 4:**
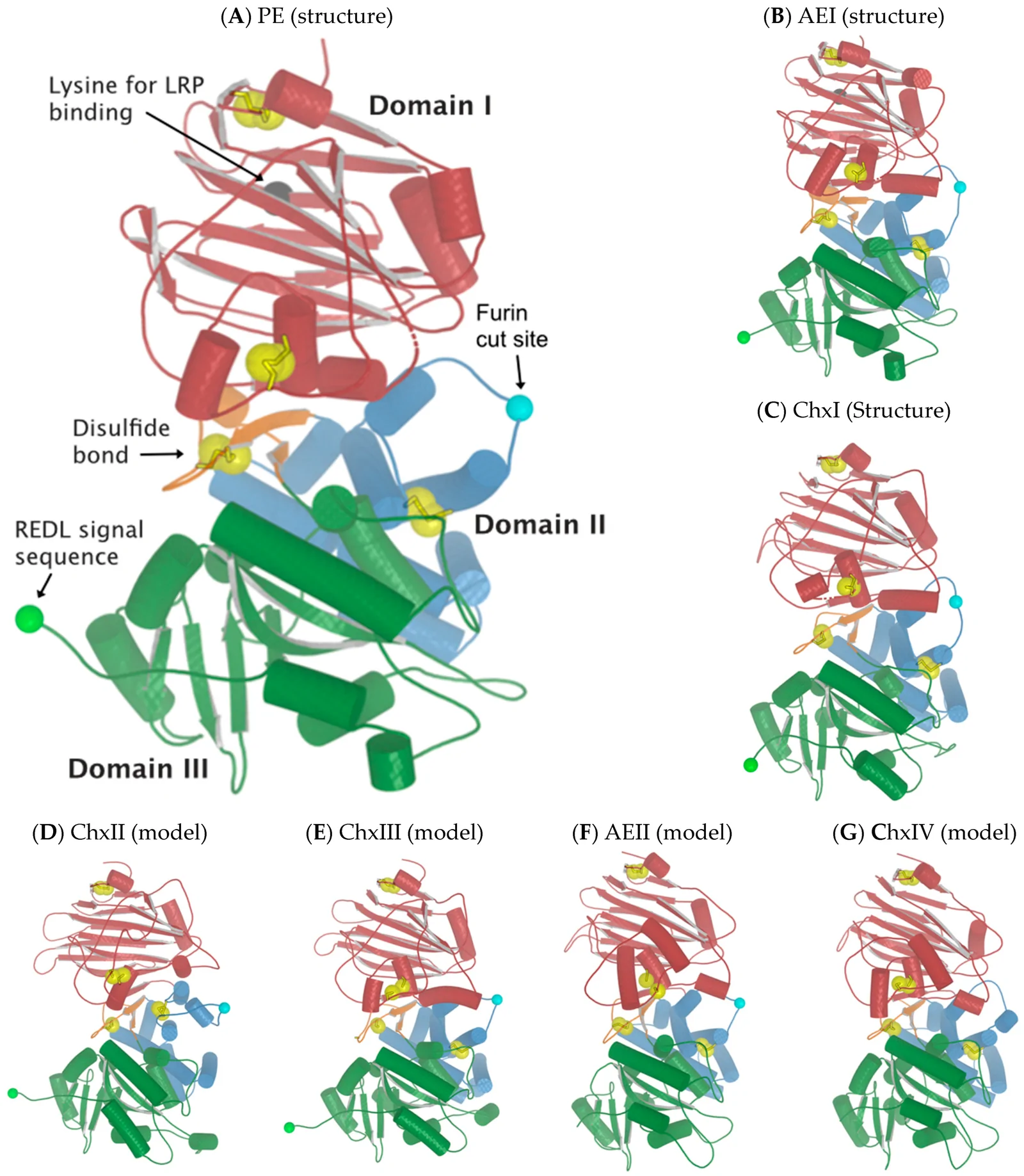
Structures of diphthamide-specific mono-ADP-ribosylating toxins. (**A**) PE crystal structure (PDB entry 1IKQ). (**B**) AEI crystal structure (PDB entry 6z5h). (**C**) ChxI crystal structure (PDB entry 2q5t). (**D**–**G**) AlphaFold models of protein sequences of ChxII, ChxIII, AEII, and ChxIV, respectively. Predicted disulfide bonds are shown as yellow spheres, predicted furin protease cleavage sites are shown in cyan, the RDEL retrograde transport C-termini are shown as light green spheres and the lysine residues important for LRP receptor binding are shown as grey spheres (present in only PE and AEI).

**Figure 5:**
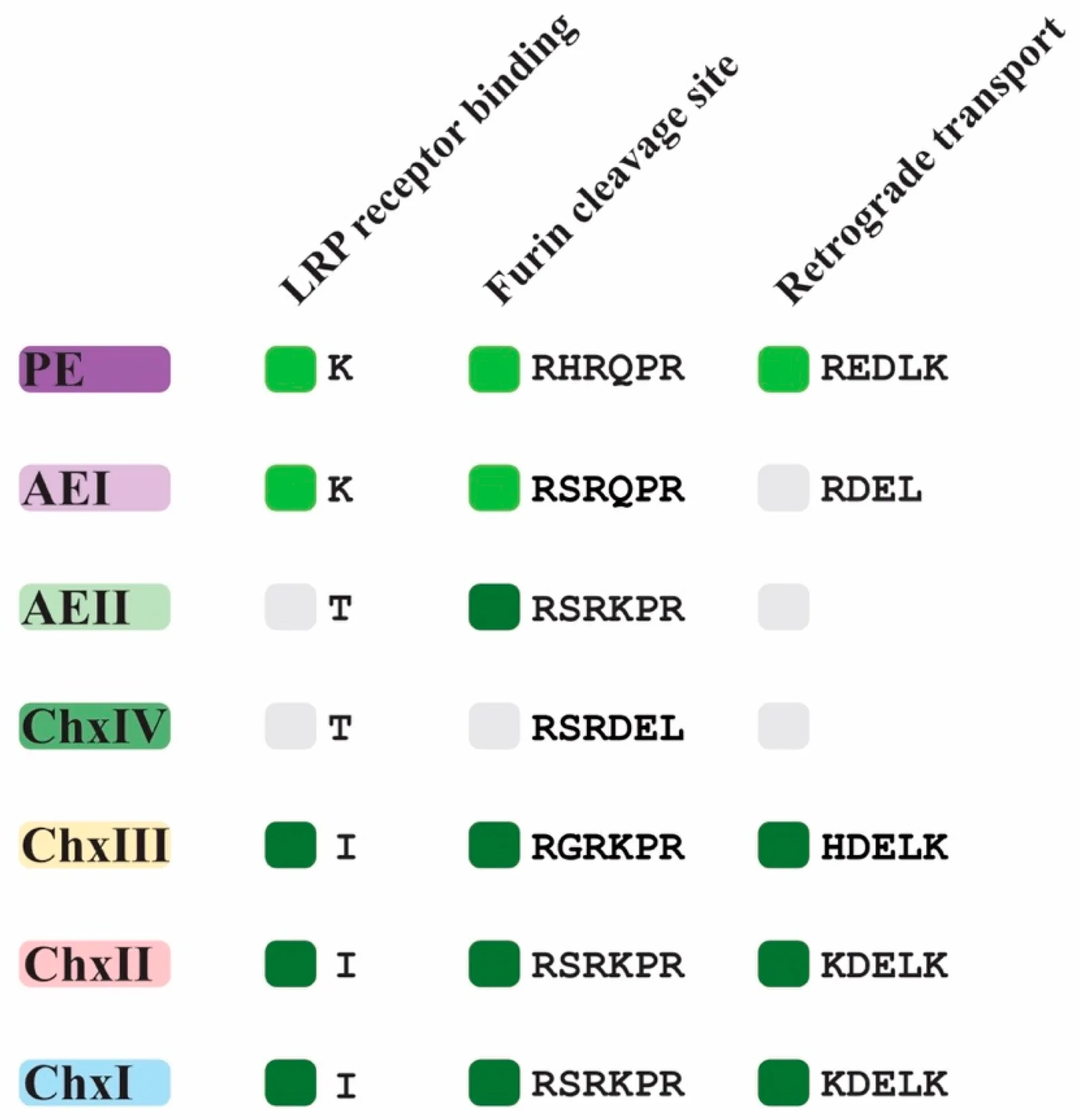
Overview of the presence or absence of key functional sites and motifs in PE, Chx-, and AE homologues. “LRP receptor binding” refers to the lysine residue (K) which has been shown to be important for receptor binding of PE [12,43,44]. “Furin cleavage site” refers to the conserved sequence motif recognized by the furin protease [13,45,46], while “Retrograde transport” is the C-terminal sequence shown to direct the toxin sequence to the endoplasmic reticulum (ER) before translocation to the cytoplasm [40]. Motifs resembling PE are represented by light green boxes, while dark green boxes indicate resemblance to ChxI. Grey boxes indicate lack of conservation or missing motifs. The motif residues are written next to the boxes. Toxin names are colored according to the phylogenetic tree in Figure 1. The functional sites and sequence features represented in this figure correspond to full sequences shown in Figure 2.

**Table 1:** Selected results of a PSI-BLAST search of NCBI with PE sequence from *Pseudomonas aeruginosa* as a query.

Scientific Name	Max Score ^c^	Coverage (%)	Identity (%) Full-Length	Identity (%) Cat. Domain	Accession Length	GenBank Accession No.
*Pseudomonas aeruginosa* ^a^		100	100	100	638	WP_058170169.1
*Aeromonas hydrophila* ^b^	1134	95	64.59	74.64	628	6Z5H_AAA
*Aeromonas bestiarum*	1124	94	64.40	75.12	608	MDM5070604.1
*Aeromonas dhakensis*	1154	96	64.19	75.60	624	UCM47367.1
*Aeromonas oralensis*	1161	98	63.17	74.19	626	WP_438834555.1
*Aeromonas salmonicida*	1131	96	62.26	71.29	624	MDQ1883297.1
*Aeromonas piscicola*	1122	97	61.58	70.33	626	WP_082030310.1
*Aeromonas sobria*	1092	97	55.97	70.33	640	MGL6150327.1

^a^ The PE query sequence used for the PSI-BLAST search (dark purple). ^b^ *Aeromonas* (AE) sequence with known crystal structure. All the selected search results (light purple) had an E-value below 1 × 10^−180^. Values for coverage and max score are for the full-length toxin search. ^c^ Max Score is the BLAST bit score, which reflects both alignment length and percent identity to the query; it was used to rank hits and select the representative sequence for each species.

**Table 2:** Selected results of a PSI-BLAST search of NCBI with the ChxI sequence from *Vibrio cholerae* as a query.

Scientific Name	Max Score ^b^	Coverage (%)	Identity (%) Full-Length	Identity (%) Cat. Domain	Accession Length	GenBank Accession No.
*Vibrio cholerae* ^a^		100	100	100	666	Q5EK40.1
*Aeromonas salmonicida*	963	97%	47.85%	49.31%	635	MDM5064611.1
*Aeromonas popoffii*	960	97%	47.77%	50.47%	634	WP_212514733.1

^a^ The ChxI query sequence used for the PSI-BLAST search (light blue). The selected search results (light green) had E-values of 0.0 (rounded-down probability below 1 × 10^−180^). Values for coverage and max score are for the full-length toxin search. ^b^ Max Score is the BLAST bit score, which reflects both alignment length and percent identity to the query; it was used to rank hits and select the representative sequence for each species.

**Table 3:** Selected results of a BLASTp search of NCBI with the AEII sequence from *Aeromonas popoffii* as a search query.

Scientific Name	Max Score ^b^	Coverage (%)	Identity (%) Full-Length	Identity (%) Cat. Domain	Accession Length	GenBank Accession No.
*Aeromonas popoffii* ^a^		100	100	100	634	WP_212514733.1
*Vibrio cholerae*	1000	100	83.20%	85.44%	643	EKF9159538.1
*Vibrio cholerae*	984	100	83.20%	85.44%	643	EJL6420669.1
*Vibrio cholerae*	980	100	83.05%	85.44%	643	EJL7976782.1

^a^ The query sequence used for the PSI-BLAST search (light green). All the selected search results (dark green) had an E-value of 0.0 (rounded-down probability below 1 × 10^−180^). Values for coverage and max score are for the full-length toxin search. ^b^ Max Score is the BLAST bit score, which reflects both alignment length and percent identity to the query; it was used to rank hits and select the representative sequence for each species.

## Data Availability

All the results in the manuscript are based on data available in a publicly accessible repository (e.g., the NCBI protein sequence database) and the accession numbers for the reported sequence entries are all clearly stated in the text.

## Supplementary Materials

The following supporting information can be downloaded at https://www.mdpi.com/article/10.3390/toxins18100416/s1: Figure S1: Phylogenetic tree of *Aeromonas* spp. sequences; Figure S2: Alignment of selected AEI N-terminal sequences; Table S1:Genomes containing co-occurring AEI and AEII sequences; Table S2: All the 425 unique sequences of predicted signal peptides.
