# Supplementary material for: Database Mining Reveals Distinct Clusters of Putative Diphthamide-Specific Mono-ADP-Ribosylating Toxins in Vibrio and Aeromonas
Source: Toxins (Basel). 2026 Sep 28;18(10):416. doi: 10.3390/toxins18100416 (PMC13709805; doi:10.3390/toxins18100416)
Supplement: Supplementary file 1 [file toxins-18-00416-s001.zip › toxins-4485157-supplementary.pdf]

Supplementary Materials: Database Mining Reveals Distinct Clusters of Putative Diphthamide-Specific Mono-ADP-Ribosylating Toxins in *Vibrio* and *Aeromonas*

Marta Sienkiewicz, Geoffrey Masuyer and René Jørgensen

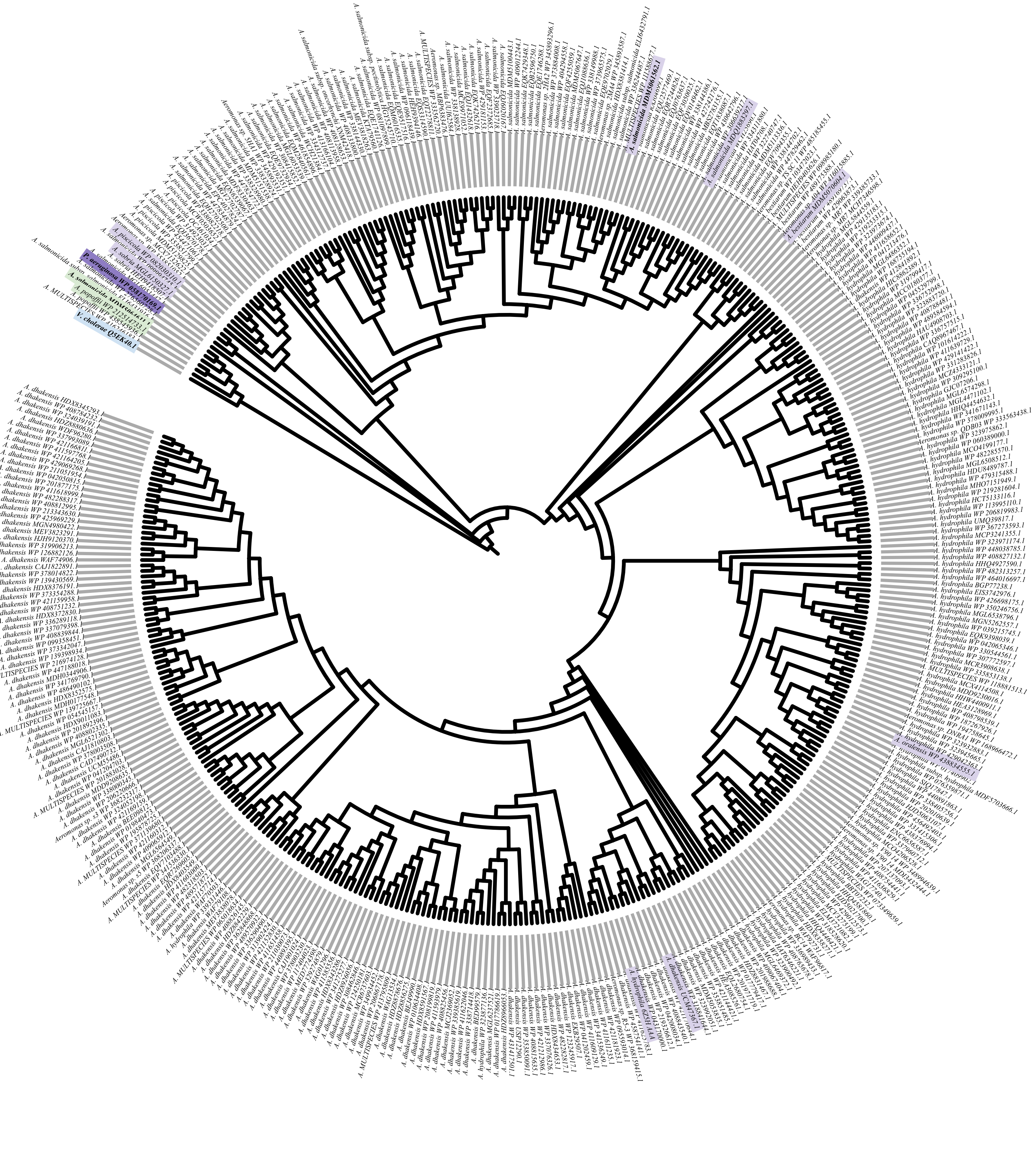

Supplementary Figure S1. Phylogenetic tree showing 398 *Aeromonas* spp. sequences identified by PSI-BLAST using PE (WP_058170169.1) as the query and 6 *Aeromonas* spp. sequences identified using ChxI (Q5EK40.1) as the query, resulting in 404 sequences after filtering (filtering criteria were 40–100% identity and 90–100% query coverage). PE (dark purple) and Chx (light blue) are shown as references. Branch lengths were ignored. The sequences highlighted in light purple and light green correspond to sequences shown in Tables 1 and 2 and Figure 1. Additionally, *A. salmonicida* (MDM5061562.1) is highlighted in light purple and marked in bold; this sequence corresponds to AEI in Figure 2.

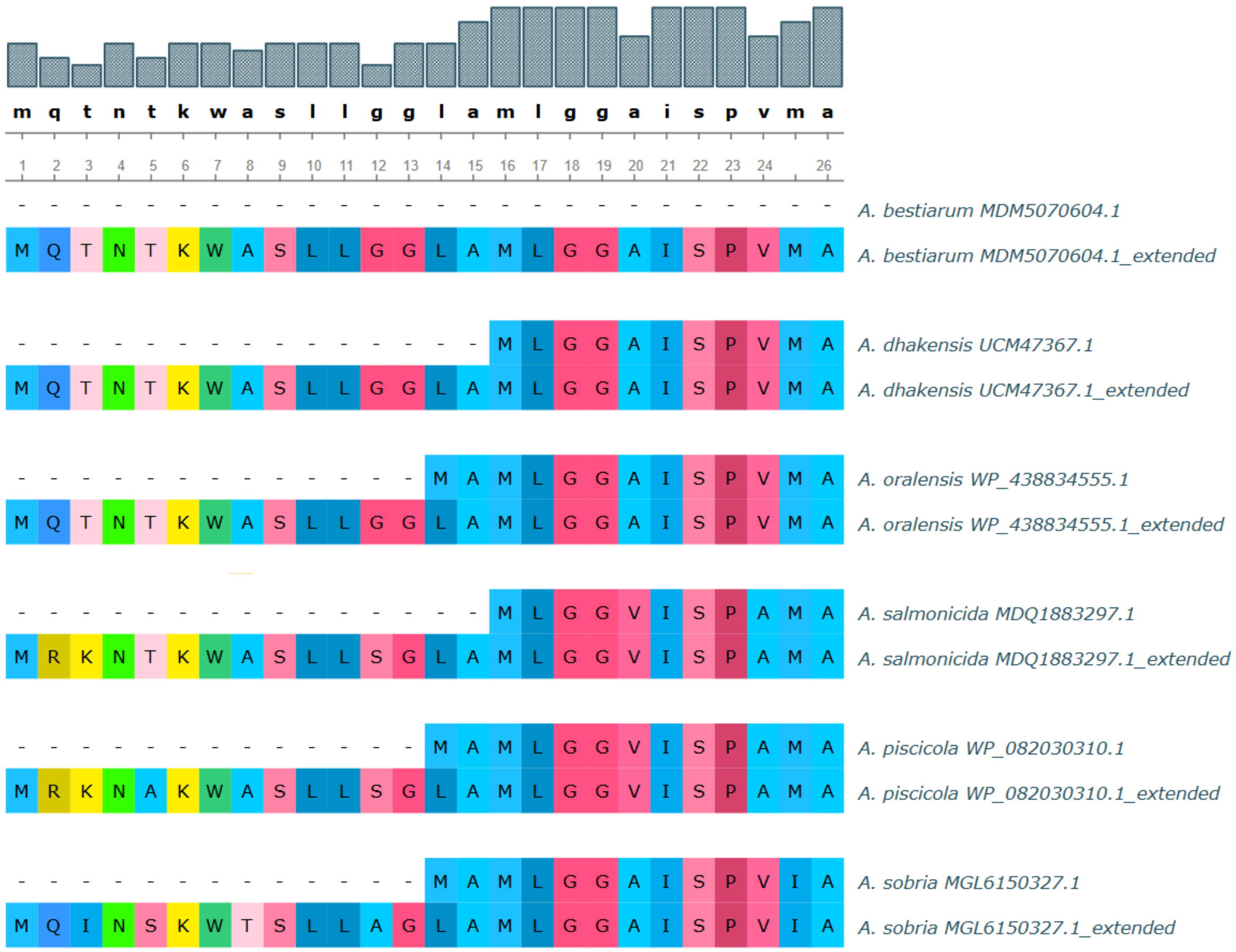

Supplementary Figure S2. Alignment of selected AEI N-terminal sequences and their extended versions. Extended versions were obtained by adding upstream sequences based on the genomic information associated with the corresponding original protein sequences. The sequences were analyzed using SignalP 6.0 to predict signal peptides. SignalP 6.0 predicted Sec/SPI signal peptides in all extended sequences, with probabilities of 0.998–0.999 for Sec/SPI and with predicted cleavage sites at positions 26–27. The original protein sequences were also predicted to contain Sec/SPI signal peptides, except for A. bestiarum MDM5070604.1, for which the extended sequence was required for signal peptide prediction. Predicted Sec/SPI probabilities for the original (shorter) protein sequences were nevertheless considerably lower. The alignment and SignalP predictions suggest that some sequences in the original protein entries may represent truncated or incorrectly annotated protein sequences.

Supplementary Table S1. Genomes containing co-occurring AEI and AEII sequences, with the corresponding protein, nucleotide, and genome assembly accession numbers.

Scientific name	GenBank accession no.	Toxin type	GenBank accession no.	Nucleotide accession	RefSeq or GenBank assembly accession
Aeromonas salmonicida	WP_338149988.1	AEI	WP_338149988.1	NZ_JAOXBX010000001.1	GCF_026173125.1
Aeromonas salmonicida	WP_276560728.1	AEII	WP_276560728.1	NZ_JAOXBX010000039.1	
Aeromonas salmonicida	MDM5061562.1	AEI	MDM5061562.1	JAOPLC010000001.1	GCA_030386575.1
Aeromonas salmonicida	MDM5064611.1	AEII	MDM5064611.1	JAOPLC010000021.1	
Aeromonas salmonicida	ELI6432791.1	AEI	ELI6432791.1	ABNAHJ010000024.1	GCA_032094795.1
Aeromonas salmonicida	ELI6431916.1	AEII	ELI6431916.1	ABNAHJ010000012.1	

Supplementary Table S2. All 425 unique sequences of predicted signal peptides. No. of accessions is the number of sequences in which a given predicted signal peptide was present.

Category	Predicted signal peptide	No. of accessions	Signal peptide length	Probability for Sec/SPI
PE	MHLISHWIPLVASLGLLAGGSFASA	1	25	0.999–0.999
AEI shorter	MAMLGGAISPVMA	233	13	0.501–0.999
	MAMLGGVISPAMA	77	13	
	MLGGAISPVMA	31	11	
	MAMLGGAISPVIA	3	13	
	MLGGVISPAMA	3	11	
	MAMLGGTISPVMA	2	13	
	MTMLGGAISPVMA	2	13	
	MAILGGAISPVMA	1	13	
	MAMLGSAISPVMA	1	13	
AEI longer	MQTNTKWASLLGGLAMLGGAISPVMA	15	26	0.999–0.999
	MRKNAKWASLLSGLAMLGGVISPAMA	12	26	
	MRKNTKWASLLSGLAMLGGVISPAMA	8	26	
	MQTNTKWASLLGCLAMLGGAISPVMA	1	26	
	MQTNTKWASLLGGFAMLGGAISPVMA	1	26	
	MQTNTKWASLLGGLAMLGSAISPVMA	1	26	
AEII	MIKLAIGSLLLFFTQQASS	2	19	0.999–0.999
	MIKLAIGSVLLFFTQQASS	2	19	
	IMIKLAIGSLLLFFTQQASS	1	20	
	MKNISIGVFLFLCFIQQAIS	1	20	
ChxIV	MKRLAIGSVLLFFTQQALS	3	19	0.999–0.999
ChxIII	MKKMLLIAGATVISSMAHPTFA	3	22	0.999–0.999
ChxII	LYLTFYLEKVMKKMLLIAGATVISSMAHPTFA	9	32	0.999–0.999
ChxI	MKKMLLIAGATVISSMAHPTFA	1	22	0.999–0.999
	MKNILLIAGATVISSMAHPTFA	1	22	
	LYLTFYLEKVMKKMLLIAGATVISSMAHPTFA	9	32	
	MYLTFYLEKVMKKMLLIAGATVISSMAHPTFA	1	32	
